# How do King Cobras move across a major highway? Unintentional wildlife crossing structures may facilitate movement

**DOI:** 10.1002/ece3.8691

**Published:** 2022-03-16

**Authors:** Max Dolton Jones, Benjamin Michael Marshall, Samantha Nicole Smith, Matt Crane, Inês Silva, Taksin Artchawakom, Pongthep Suwanwaree, Surachit Waengsothorn, Wolfgang Wüster, Matt Goode, Colin Thomas Strine

**Affiliations:** ^1^ 65162 School of Biology Suranaree University of Technology Nakhon Ratchasima Thailand; ^2^ School of Bioresources and Technology King Mongkut’s University of Technology Thonburi Bangkok Thailand; ^3^ Center for Advanced Systems Understanding (CASUS) Görlitz Germany; ^4^ Helmholtz‐Zentrum Dresden‐Rossendorf (HZDR) Dresden Germany; ^5^ Thailand Institute of Science and Technological Research Nakhon Ratchasima Thailand; ^6^ 1506 Molecular Ecology and Fisheries Genetics Laboratory School of Natural Sciences Bangor University Bangor UK; ^7^ School of Natural Resources and Environment University of Arizona Tucson Arizona USA

**Keywords:** bridge, drainage culvert, mortality, *Ophiophagus hannah*, road crossing, space use

## Abstract

1. Global road networks continue to expand, and the wildlife responses to these landscape‐level changes need to be understood to advise long‐term management decisions. Roads have high mortality risk to snakes because snakes typically move slowly and can be intentionally targeted by drivers.
2. We investigated how radio‐tracked King Cobras (*Ophiophagus hannah*) traverse a major highway in northeast Thailand, and if reproductive cycles were associated with road hazards.
3. We surveyed a 15.3 km stretch of Highway 304 to determine if there were any locations where snakes could safely move across the road (e.g., culverts and bridges). We used recurse analyses to detect possible road‐crossing events, and used dynamic Brownian Bridge Movement Models (dBBMMs) to show movement pathways association with possible unintentional crossing structures. We further used Integrated Step Selection Functions (ISSF) to assess seasonal differences in avoidance of major roads for adult King Cobras in relation to reproductive state.
4. We discovered 32 unintentional wildlife crossing locations capable of facilitating King Cobra movement across the highway. While our dBBMMs broadly revealed underpasses as possible crossing points, they failed to identify specific underpasses used by telemetered individuals; however, the tracking locations pre‐ and post‐crossing and photographs provided strong evidence of underpass use. Our ISSF suggested a lower avoidance of roads during the breeding season, although the results were inconclusive. With the high volume of traffic, large size of King Cobras, and a 98.8% success rate of crossing the road in our study (nine individuals: 84 crossing attempts with one fatality), we strongly suspect that individuals are using the unintentional crossing structures to safely traverse the road.
5. Further research is needed to determine the extent of wildlife underpass use at our study site. We propose that more consistent integration of drainage culverts and bridges could help mitigate the impacts of roads on some terrestrial wildlife.

## Graphical abstract

Unintentional wildlife crossing structures (drainage culverts and bridges) appear to facilitate the safe movement of King Cobras (*Ophiophagus hannah*) across a major highway in Thailand. We recorded a 98.8% success rate for King Cobras crossing the road, suggesting that our telemetered snakes are using some of the 32 crossing structures discovered in our study area to achieve this. Further research should investigate the true propensity for terrestrial wildlife to use these structures, which may warrant more frequent integration into road construction globally.

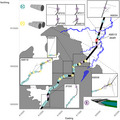

## 1 INTRODUCTION

Southeast Asia is one of the world's many biodiversity hotspots, combining a rich fauna and flora with a myriad of human threats that are endangering the biodiversity (Hughes, 2017; Myers et al., 2000; Ng et al., 2020). The growing human population in Southeast Asia continues to increase urbanization (Schneider et al., 2015), and road networks continue to expand to meet human demands, posing threats to wildlife (Ascensão et al., 2018; Hughes, 2018). Roads are either diffuse or hard barriers to wildlife movement (Brehme et al., 2013; Shepard et al., 2008), dividing habitats and resources, and potentially undermining wildlife population integrity and reducing genetic diversity (Aresco, 2005; Balkenhol & Waits, 2009; Clark et al., 2010; Herrmann et al., 2017; Jackson & Fahrig, 2011; Row et al., 2007). Alongside fragmenting habitats, roads can also constitute a direct source of mortality for wildlife via vehicular collisions (Aresco, 2005; Bernardino & Dalrymple, 1992; Das et al., 2007; Lodé, 2000; Rosen & Lowe, 1994; Row et al., 2007). Some drivers can intentionally target certain animals, such as snakes; therefore, potentially leading to targeted species being disproportionately affected by roads (Langley et al., 1989; Ashley et al., 2007; Beckmann & Shine, 2012; Assis et al., 2020; but not detected by Secco et al., 2014).

Wildlife managers can alleviate the risks from roads, mitigate road mortality, and facilitate animal movement by implementing wildlife‐crossing infrastructure (Lister et al., 2015). Once wildlife becomes acclimated to crossing structures, the structures help sustain animal mobility across fragmented landscapes, aiding wildlife access to resources and conspecifics for gene flow (Clevenger & Barrueto, 2014). Wildlife‐crossing locations can be underpasses, such as culverts or tunnels, or overpasses, which are generally large vegetated land bridges (Clevenger & Huijser, 2009; Dodd et al., 2004; Glista et al., 2009). Wildlife crossings have been successful in facilitating animal movement in several cases (Beckmann et al., 2010; Forman, 2003), such as for mule deer in western North America (Simpson et al., 2016), a diversity of wild mammals in Poland (Myslajek et al., 2016; Ważna et al., 2020), and turtle species in Canada (Markle et al., 2017).

Snakes are common victims of roads, likely due to low movement speeds, unique body shape, and mode of locomotion (Andrews & Gibbons, 2005). Additionally, snakes are disproportionately targeted by road users (Ashley et al., 2007; Beckmann & Shine, 2012), and thus an important group to protect from the risks presented by roads. However, studies so far reveal an infrequent, and unpredictable, use of ecopassages by snakes (Baxter‐Gilbert et al., 2015). Additionally, fencing and other man‐made barriers are essential structures needed to direct reptile species to ecopassage locations and are often the only mitigation strategy which prevents these species from accessing the roads surface (Baxter‐Gilbert et al., 2015). Researching the propensity for snakes to use ecopassages without these directive barriers can provide invaluable information for wildlife management.

The King Cobra (*Ophiophagus hannah* [CANTOR, 1836]), is a large, venomous snake widely distributed throughout Southeast Asia and ranging from India to China and the Philippines. The IUCN classifies King Cobras as Vulnerable (Stuart et al., 2012), with decreasing populations and urges investigations into specific threats. Andrews and Gibbons (2005) showed that stout‐bodied species (in the Southeastern USA) had slower crossing speeds than longer, slender‐bodied sympatric species. This suggests that King Cobras could also exhibit relatively fast movement speeds across roads; however, these crossing speeds would likely be undermined by the large length and mass of this active forager. Actively foraging species, with high mobility, demonstrate plasticity in their use of microhabitats (such as the use of open areas or road‐adjacent microhabitats), often increasing their risk from roads (Forman, 2003; Hartmann et al., 2011; Paterson et al., 2019). King Cobras are susceptible to road mortality in areas where major roads divide habitats, such as in the Sakaerat Biosphere Reserve (SBR) in northeast Thailand (Marshall et al., 2018, 2020). Despite small sample sizes of mortalities in Marshall et al. (2018), four vehicle collisions were recorded among a total of 14 mortality events, prompting further investigation into the potential impacts roads have on King Cobras.

During routine road construction, plans typically integrate drainage culverts sporadically to divert water from road surfaces. However, such structures may also act as unintended wildlife‐crossing locations for small taxa (Aresco, 2005; Ascensão & Mira, 2007; Brunen et al., 2020; Clevenger & Waltho, 2000; Grilo et al., 2008; Ng et al., 2004; Sparks & Gates, 2017). In central Ontario, Canada, Baxter‐Gilbert et al. (2015) found that three reptile species used culverts as ecopassages during monitoring: Painted Turtles (*Chrysemys picta*), Snapping Turtles (*Chelydra serpentina*), and Northern Watersnakes (*Nerodia sipedon*). In addition, Aresco (2005) demonstrated the importance of an under‐highway culvert for reducing turtle mortality, when augmented with drift fences.

Based on the evidence presented above, it is possible that King Cobras are using unintended wildlife crossing structures to safely traverse the roads. The abundance and importance of roads in and around the SBR make this an ideal site to explore the role of these structures in assisting movement across major roads by King Cobras. Using a long‐term dataset on the movement ecology of King Cobras in northeast Thailand, we addressed the following questions: (1) Are there any structures present along the highway which could facilitate King Cobra movement? (2) Is there evidence suggesting that King Cobras are using structures to cross the highway as opposed to moving over the roads surface? and (3) Are King Cobra reproductive cycles associated with road hazards (e.g., seasonal avoidance of roads or increased rates of vehicle collision)?

## 2 METHODS

### 2.1 Study site

**FIGURE 1:**
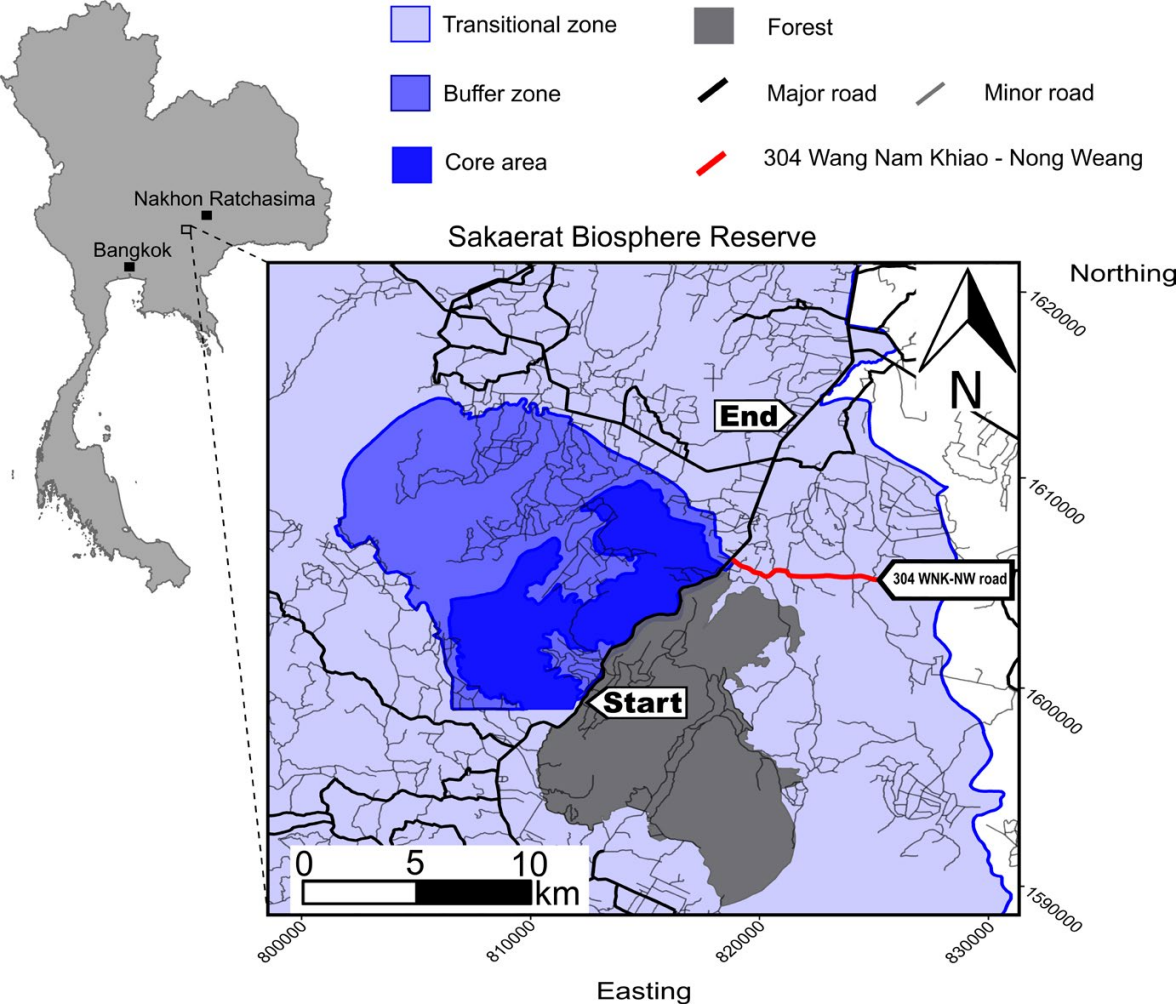
Study site map in relation to Bangkok and Nakhon Ratchasima cities. The three Sakaerat Biosphere Reserve zones are delineated by their level of protection via increased opacity (increasing opacity represents increased protection). The bold red line shows the 304 Wang Nam Khiao–Nong Weang road. The *Start* and *End* mark the section of Highway 304 included in our study

We conducted field work from 22 March 2014 to 28 August 2020, at the Sakaerat Biosphere Reserve (SBR), Nakhon Ratchasima Province, Thailand (14.44–14.55°N, 101.88–101.95°E; Figure 1). The SBR consists of three areas of differing levels of protection: a core protected forested area covering 80 km^2^ consisting of mainly dry dipterocarp forest and dry evergreen forest; a buffer zone consisting of areas of reforestation and plantations; and lastly a transitional area dominated by agriculture (rice, cassava, corn, and sugar). The transitional area contains 159 settlements with 72,000 inhabitants (Thailand Institute of Scientific and Technological Research, 2018), and a network of both paved and dirt roads. The forested areas of the core area and transitional zone are bisected by the National Highway 304 (with dense, expansive forest on either side of the highway), built initially in 1956, with further road improvement in 1966 and subsequent expansion from two to four lanes in 2005 (Laurence, 2014; Vaeokhaw et al., 2020). Highway 304 transects several protected forests, including the largest fragment of surviving forest in Central Thailand, that host high biodiversity of threatened or endangered herpetofauna (Silva et al., 2020).

A second major road perpendicular to the Highway 304, Wang Nam Khiao–Nong Weang (304WNK‐NW), passes through a populated area in the transitional zone, east of the Highway 304 (Figure 1). We studied this road because it is the first major road (with tarmac and multiple lanes) separating the agricultural area from the unprotected forest fragment to the south; therefore, the road has substantial conservation implications, especially for female King Cobras that are forced to circumvent the road to reach forested areas, where oviposition typically occurs (Marshall et al., 2020).

### 2.2 Capture

We captured King Cobras between March 19, 2014 and March 18, 2020, using a combination of opportunistic captures, villager notations, and active visual surveys (Marshall et al., 2018, 2019, 2020). We gave each snake a unique ID based on age class, sex, and capture number (e.g., AM018 refers to an adult male that was the 18th King Cobra captured, JF055 refers to a juvenile female that was the 55th King Cobra captured).

We anesthetized King Cobras using isoflurane to obtain accurate morphological measurements and perform radio‐transmitter implant surgeries, following procedures outlined in Reinert and Cundall (1982). We implanted Holohil AI‐2T or SI‐2T transmitters into the coelomic cavity. We initially marked individuals with a unique brand on the ventral/dorsal scales using a disposable medical cautery device (Winne et al., 2006). We switched to passive integrated transponders (PIT‐tags) beginning with AM054.

We released snakes within 24 h post‐surgery, at an average distance of 191 m (range = 0–1,263 m, Table S1) from their capture site. We aimed to release snakes as close to their reported capture site as possible; however, distances had to be increased when snakes were captured within or near homes. Our largest recorded distance released from capture location was the result of needing to remove the snake from a large human settlement area, as requested by residents. Release locations were not considered as telemetered locations, and therefore not included in any subsequent analyses, but were shown to be within the estimated occurrence distributions of our telemetered individuals. Because we recaptured AM006, AM007, and AF010 after transmitters from their first implant failed, we used capture and release information from this subsequent recapture (Table S1).

### 2.3 Radio telemetry

Radio‐tracking protocol changed throughout the study due to staff availability and changes in investigation targets (initially, we only aimed to assess home range sizes and habitat use, while in later years we added movement patterns and site fidelity to the program). We tracked snakes nearly continuously, until the death of AM005 (Strine et al., 2014), and maintained signal contact with the snakes every 15 min, determining a snake's location every hour between 6:00 a.m. and 10:00 p.m. From March 9 2014 to July 28 2018, we radio tracked individuals 006–026 four times per day (i.e., 6:30 a.m., 11:00 a.m., 4:00 p.m., 10:00 p.m.), with a mean of 8.5 SE ± 0.1 h between fixes. We radio tracked individuals 027–099, from July 28, 2018, to August 1, 2020, three times per day aiming for 5 h intervals between successful pinpoints (Figure S1 displays overall study time lag distribution for each individual). We usually radio‐tracked King Cobras in daylight; however, we occasionally radio tracked snakes at night, depending on individual movements and landscape type. We triangulated snake locations, attempting to maintain a minimum distance of 10 m from the snake (occasionally compromised by sub‐10 m GPS accuracy or difficult terrain), which enabled us to be reasonably confident that the snake was within a 5 m^2^ area. We recorded the triangulated location (Universal Transverse Mercator 47 P WGS 84 datum) using handheld GPS units, recording date, time, and GPS accuracy.

### 2.4 Quantifying crossing structure characteristics

We identified drainage culvert locations along Highway 304 using roadside markers, presumably set by construction workers. No over‐the‐road structures exist at our study area, and therefore all crossing structures mentioned herein refer to corridors which allow animals to move directly underneath the road. We recorded locations of both entrances for all drainage culverts and bridges (viaducts) encountered, along with vertical diameter of entrance (mm), horizontal diameter of entrance (mm), length of structure (m), vegetation cover at entrance (yes/no), dominant substrate within the structure, and connectivity to landscape feature (i.e., none, stream, or irrigation canal) for each crossing. We calculated distances (m) between adjacent potential crossing structures with the measuring tool in QGIS (v. 3.14.15 “pi”).

### 2.5 Identifying road‐crossing events

We manually created spatial polygons using QGIS v.3.14.15 “pi”, for the entire study area encompassing the side of Highway 304 that contained the core protected area, herein referred to as *North Side* (Figure S2). We used the *recurse* package v.1.1.0 in R (Bracis et al., 2018) to calculate the number of times each telemetered snake entered, or exited, the North‐Side spatial polygon, which corresponded to a road‐crossing event across Highway 304 (Figure S2). We also created a spatial polygon encompassing the area south of the 304WNK‐NW road; herein, referred to as *South Side* (Figure S3). Using the *recurse* package, we recorded each time a nesting female King Cobra entered, or exited, the South‐Side spatial polygon, corresponding to an event during which the snake traversed the road (Figure S3). Due to only having one adult male which interacted with the 304WNK‐NW road and poor temporal resolution for this individual, we chose to only sample adult female King Cobras for the *South*‐*Side* spatial polygon. This allowed us to investigate if there were any temporal patterns for how reproductive females interact with the 304WNK‐NW road during nesting movements.

The recurse analysis provided the approximate time that a highway crossing event occurred for each snake (although the time provided is restricted to the nearest data point collected). We then took subsets of the radio‐tracking data that consisted of fixes taken 2 weeks prior to, and 2 weeks after, each crossing event. We ran dynamic Brownian Bridge Movement Models (dBBMMs) on these subsets to estimate an occurrence distribution describing the uncertainty associated with potential movement pathways during a crossing event, using the *move* package v.3.1.0 in R (Kranstauber et al., 2020). Because the subsets were shorter time periods than our overall tracking periods, we used a window size of 15 and margin size of 3 to detect temporally fine‐scale changes in movement states (specifically, shifts between resting/sheltering and movement) when using underpasses (i.e., the number of data points over which the snake's movement capacity was estimated over; see Smith et al., 2021, for a further concise explanation of window and margin size). Following the methods outlined in Marshall et al. (2020), we extracted 90%, 95%, and 99% contours (confidence areas), using R packages *adehabitatHR* v.0.4.16 (Calenge, 2006), and *rgeos* v.0.4.2 (Bivand & Rundel, 2020), to visualize the movement pathways when crossing Highway 304. We visually inspected each dBBMM subset of crossing events to discern if the extracted contours, representing possible movement pathways, contained a crossing structure. If a crossing structure was observed within the 99% and 90% contour, we extracted new contours decreasing in increments of 10%, and made further visual checks for crossing structure presence within the contours. The minimum contour we extracted was 20% due to visual accuracy. Examples of our dBBMM subsets can be seen in Figures S4–S10. In addition to our dBBMM subsets, we also visualized the points directly before and after a crossing event to investigate if single, direct movements could also serve as a proxy to determine underpass use by adult King Cobras.

### 2.6 Integrated step‐selection function

We used Integrated Step‐Selection functions (ISSF) from the *amt* package v.0.0.6 in R (Signer et al., 2019) to assess the influence of major roads on adult King Cobra movement (i.e., avoidance or association). Integrated Step‐Selection functions use observed locations (steps) of telemetered animals to generate random steps using observed step characteristics (step length and turning angle). In our ISSF analyses, we investigated if Euclidean distance to major roads (log‐transformed), step length, and turning angle differed between observed (actual) steps and randomly generated steps, between two species‐relevant seasons (outlined below).

We separated tracking periods into two seasons, incorporating breeding and nesting in one season and the remainder of an individual's tracking duration during the non‐breeding season. The earliest date we observed breeding (over multiple years) was March 10, and the latest date that a female left her nest was July 05, which we used to define the extent of breeding season. Due to the tracking regime, and therefore resolution of our data, we were unable to determine the extent of breeding season for each year. We therefore added a 10‐day buffer to the start and end dates to account for natural variation that we may have missed in the population, which conveniently resulted in an annual breeding season from March 1 to July 15. We used an inverted raster layer (10 m resolution) which described varying distances from major roads within the SBR; we inverted the raster to aid interpreting model outputs (Marshall et al., 2020). Following methodology by Fortin et al. (2005), Marshall et al. (2020), and Smith et al. (2021), we simulated 200 random points for each step using default distributions (i.e., fitting the step length with a Gamma distribution, and the turn angle with a von Mises distribution; Signer et al., 2019). We opted to use a large number of random points due to the coarseness of VHF radio telemetry compared to GPS telemetry data, the latter usually only affording a single, or very few, random steps per used step due to the high temporal resolution of data and computational cost (Northrup et al., 2013; Thurfjell et al., 2014). Two‐hundred random points also facilitate coverage of rare features or smaller changes within a landscape.

We describe our ISSF analyses using two broad terms: avoidance and association. These terms are used throughout to describe the proximity of observed steps (true movement locations) to major roads. *Avoidance* describes movements at a greater distance from major roads, and *association* describes movements nearer to major roads; by telemetered King Cobras. We use these terms to ease interpretation of our results, and do not use them to infer behavioral responses to the presence of major roads. We evaluated avoidance of, and association with, roads by telemetered adult King Cobras at both the individual and population levels. We opted to perform both individual‐ and population‐level models due to our small sample size, which only allows us to make limited inferences about the overall population. Each model included step length, turning angle, and (inverted) distance from major roads as predictors. We investigated population‐level effects following R script by Muff et al. (2020b), which involved using a Poisson regression model with stratum‐specific effects, and accounting for the data's structure (both individual ID and step/strata) using Gaussian processes (see Muff et al., 2020a, for detailed examples of reformulating conditional logistic regression as Poisson regression). Following Muff et al. (2020), we used fixed prior precision of 0.0001 for strata‐specific effects and fitted the model using the *INLA* v.20.03.17 package (Rue et al., 2009). We radio tracked AF010, AF096, and AF099 only during the breeding season, so these snakes were only included in the breeding models. In contrast, we radio tracked AF056 only during the non‐breeding season and therefore was only used in non‐breeding season models. We included AF017, AF058, and AF086 in both non‐breeding and breeding models. In summary, we included four adult females in population‐level non‐breeding models and six adult females in population‐level breeding models. All adult males (eight) had sufficient data to be included in all ISSF models.

## 3 RESULTS

### 3.1 Radio telemetry

From March 22, 2014, to July 28, 2020, we radio tracked 21 King Cobras: eight adult males, seven adult females, four juvenile males, and two juvenile females (Table S2). We recaptured, and subsequently radio tracked, three snakes (i.e., AM006, AM007, and AM010), after 842, 1405, and 280 days missing from the study, respectively. We radio tracked snakes for an average of 344.53 ± 55.65 days (range = 134–3,122 days). We obtained an average of 920 ± 157 fixes (range = 66–1,176 fixes) on telemetered King Cobras, with an average of 9 ± 0.06 h (range = 0.05–793.85 h; Figure S1) between fixes. Snakes relocated (moved from one location to another) on average 263 ± 48 times during telemetry (range = 31–985 relocations).

### 3.2 Road‐crossing location and characteristics

We recorded 32 potential road‐crossing locations (underpasses) along the 15.3 km section of highway (Euclidean distance from the first and last crossing point; Figure 3). Of the 32 road‐crossing locations, 21 were single drainage culverts, 7 were double drainage culverts (two culverts side by side), and 4 were bridges (Figure 3). Twenty‐six of these crossing points (24 drainage culverts and 2 bridges) were within an 8 km section of the highway adjacent the forest comprising the protected area of the SBR (Figure 3). Road‐crossing structures were spaced out along the highway at a mean distance of 536.3 ± 88.4 m (range = 191–2620 m).

Crossing structures had a mean length of 40.94 ± 1.75 m (range = 26–82 m), a mean entrance height of 1,138.16 ± 127.13 mm (range = 194–3,000 mm), and a mean entrance width of 3,792.22 ± 1,418.51 mm (range = 543–30,000 mm; Table S2). All crossing points were concrete constructions, except for one metal drainage culvert (C24). There was usually no substrate within structures (*n* = 17), although those with substrate consisted of gravel (*n* = 5), rocks (*n* = 4), water (*n* = 3), soil (*n* = 2), and in one instance a tar‐like substance. Most crossing structures were not connected to any further water flow systems (*n* = 20); nine were adjacent to stream beds and three had connecting irrigation canals (three out of the four bridges were connected to irrigation canals). All crossing structures contained some anthropogenic waste either at the entrance, or within the structure. The entrances of only four culverts were devoid of any vegetation cover (C4, C6, C14, and C28).

### 3.3 Road crossing

**FIGURE 2:**
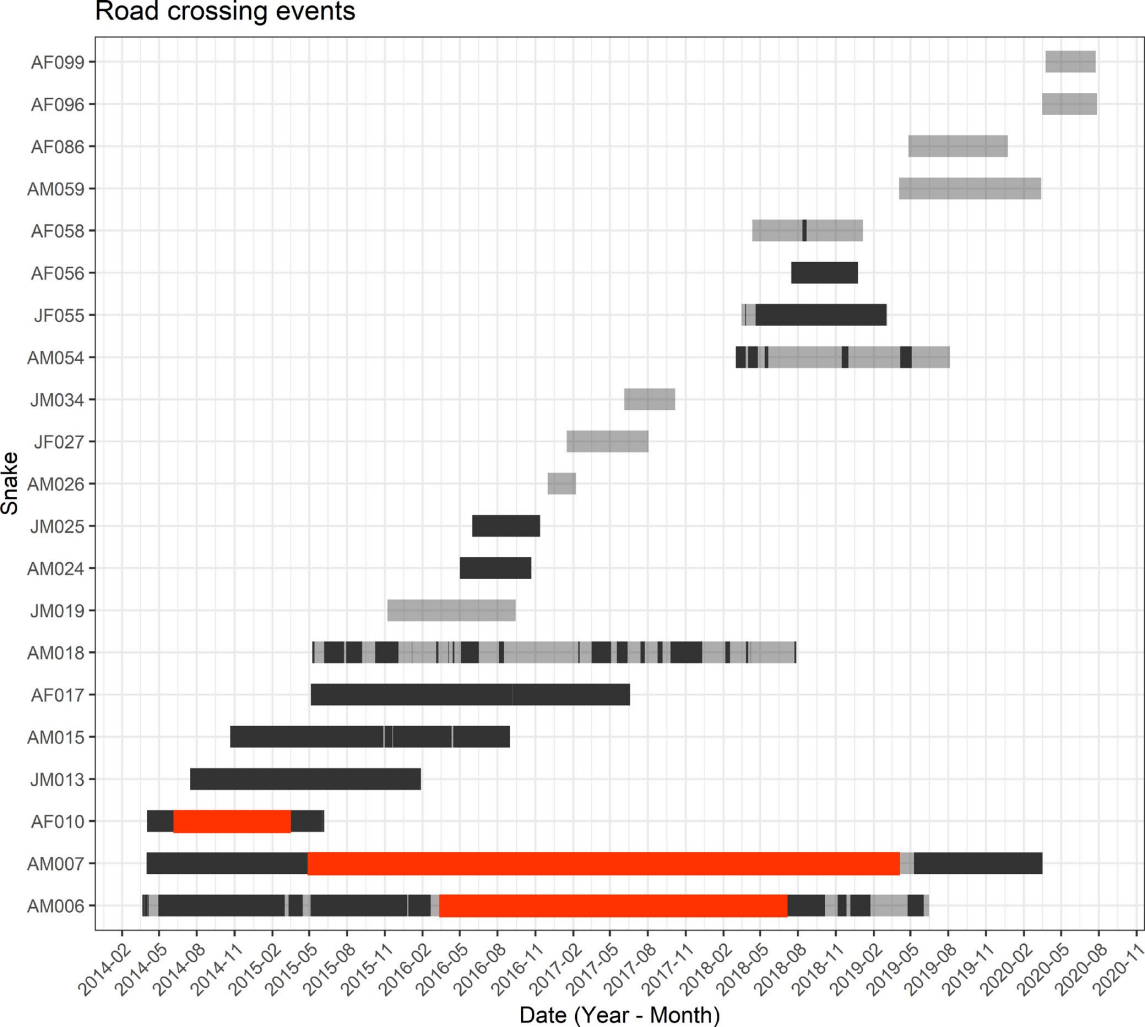
Road‐crossing events from all 21 telemetered King Cobras. Each grey bar corresponds to an individual, and opaque bars show when individuals were within the North‐Side spatial polygon. Transitions from grey to black correspond to a snake crossing over Highway 304. Red bars indicate periods of time when individuals were not radio tracked

**FIGURE 3:**
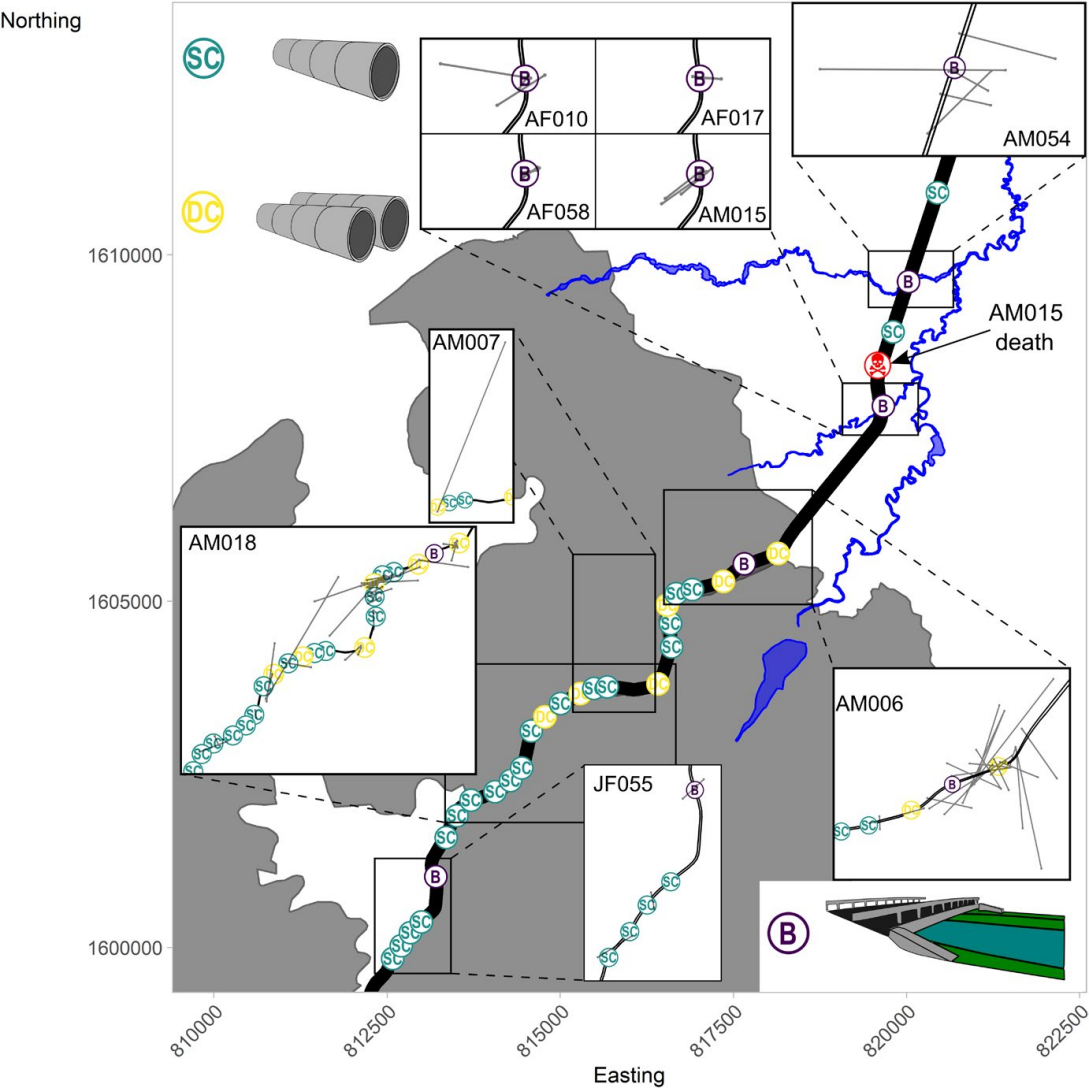
All recorded King Cobra road crossings along Highway 304 overlapped with crossing structure locations and structure type. *SC* = single culvert, *DC* = double culvert, *B* = bridge. Grey depicts forested areas and blue indicates irrigation canals and water features throughout the site. Culverts are named in chronological order (C1–C32) from southwest to northeast (Table S2). The map also depicts the location of AM015’s death (roadkill)

We confirmed 9 of 21 telemetered King Cobras had crossed Highway 304: five adult males (AM006, AM007, AM015, AM018, and AM054), three adult females (AF010, AF017, and AF058), and one juvenile female (JF055; Figure 2). Adult males crossed the highway 14 times on average (range = 1–37 times per individual), adult females crossed an average of twice (range = 2–3 times per individual), and the single juvenile female crossed four times. We ultimately recorded 84 crossing attempts of Highway 304, with one King Cobra fatality; thus, resulting in a 98.8% success rate when attempting to traverse the road (Figure 3). We could not discern any diurnal or nocturnal patterns to movement due to our sampling bias of predominantly daylight telemetry.

Via visual inspection of the dBBMM subsets produced for road crossings, we discovered that each successful crossing attempt had an underpass located within the 99% contour. Additionally, the only crossing attempt which did not contain any crossing structures within the 99% contour resulted in the death of the individual (Figure S10). The presence of crossing structures within contours decreased linearly with each 10% decrease in contour size (Table S3).

**FIGURE 4:**
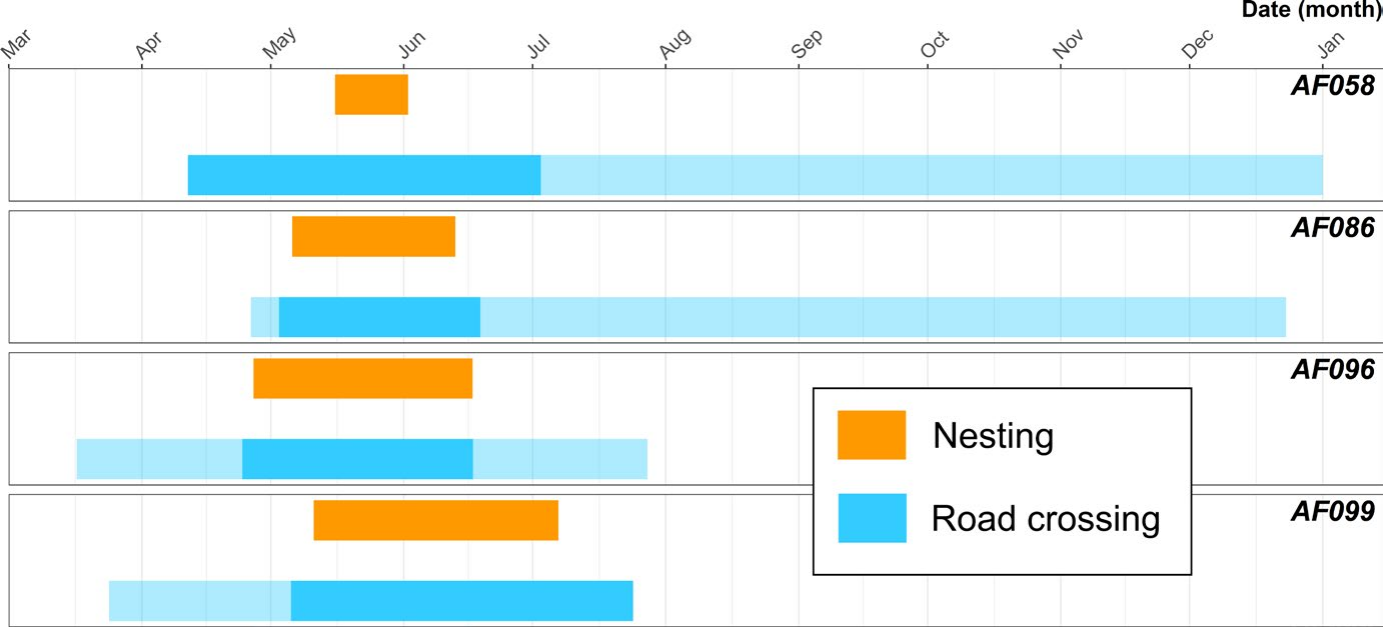
Adult female King Cobra nesting and road crossing. Orange bars highlight the nesting duration. Opaque blue bars represent when an individual was within the South‐Side spatial polygon (south of 304WNK‐NW road within forested areas for nesting). Transitions from translucent to opaque blue bars show road‐crossing events

Each telemetered female that nested on the south side of the highway crossed over 304WNK‐NW at least once during the study. All nesting females south of the road entered the South‐Side spatial polygon, and associated forested/forest‐adjacent area, between April 11 and May 5. Three of four telemetered females subsequently left the South‐Side spatial polygon, which corresponds to moving away from forested area and into the agricultural matrix, from June 18 to July 2. One female, AF099, moved north following successful nesting, making continuous movements toward 304WNK‐NW road; however, her transmitter failed on July 24, 2020, before we could observe her crossing the road and leaving the forest. We radio tracked three females, AF058, AF086, and AF096, for 182, 188, and 40 days, respectively, after they crossed back to the north side of 304WNK‐NW road. During this subsequent radio tracking, females used the agricultural landscape and we recorded no further crossing events (Figure 4).

### 3.4 Integrated step‐selection functions

**FIGURE 5:**
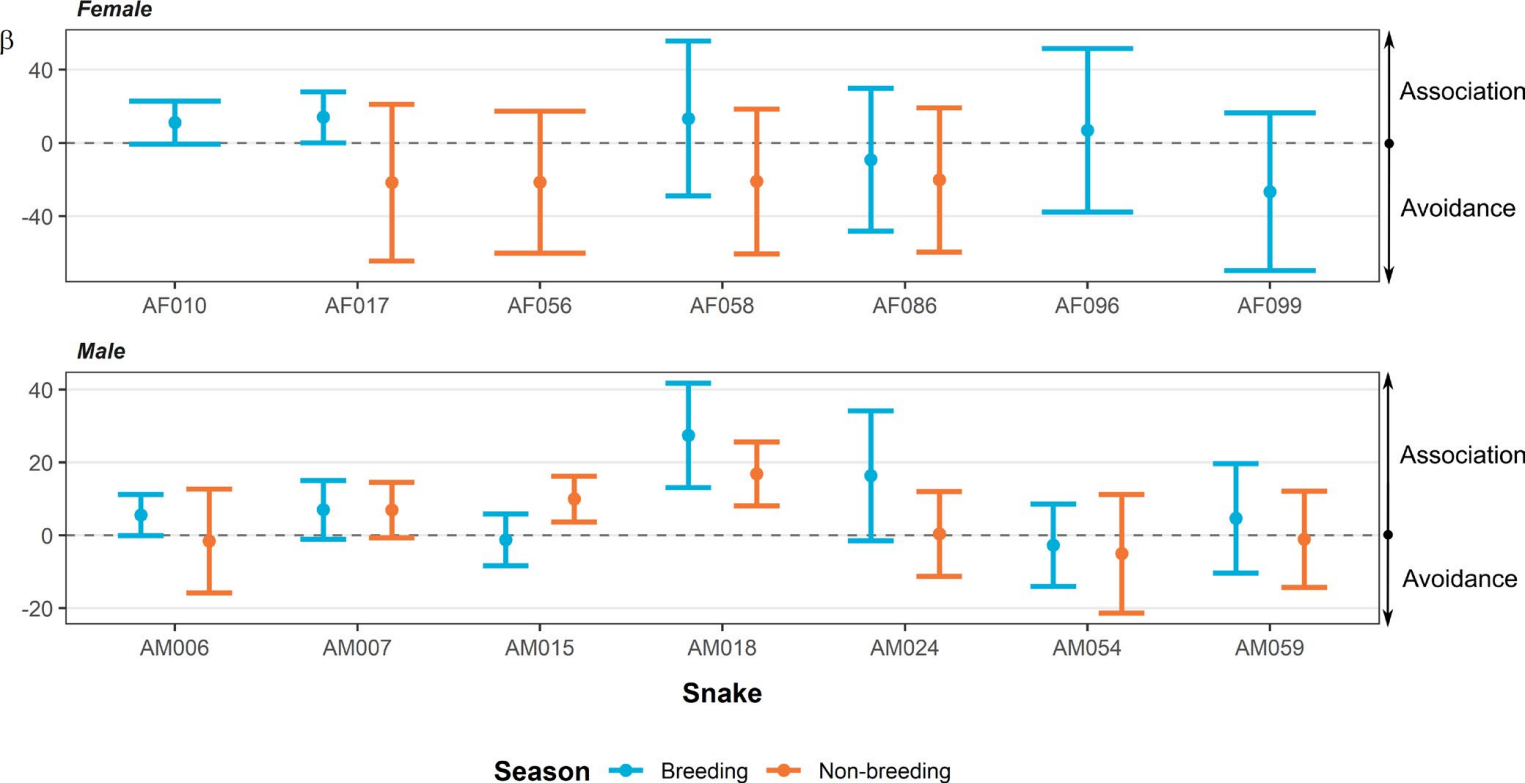
The coefficients relating to major roads from the integrated step‐selection function analyses. Breeding and non‐breeding season are depicted by blue and orange, respectively. Circles show the relative selection strength (*β*; coefficient estimate) from the model and error bars show the associated 95% confidence intervals for each estimate

**FIGURE 6:**
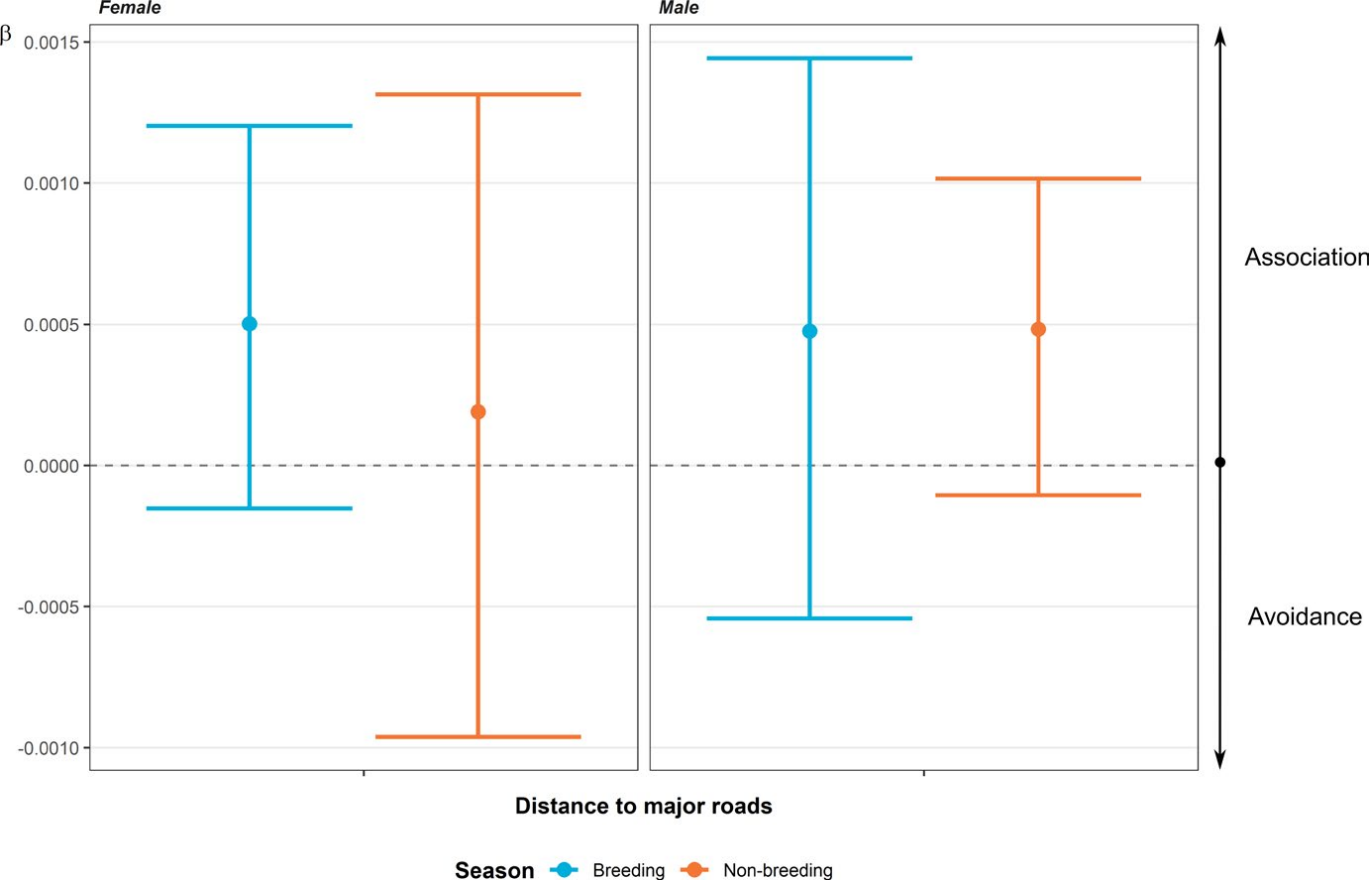
The coefficients relating to major roads from the population‐level integrated step‐selection function analyses. Breeding and non‐breeding season are depicted by blue and orange, respectively. Circles show the relative selection strength (*β*; coefficient estimate) from the model and error bars show the associated 95% confidence intervals for each estimate

Because we inverted our raster layers (Euclidean distances to major roads), positive coefficients expressed an association with roads (i.e., a closer proximity to roads). Locations of nine adult King Cobras were positively associated with major roads during the breeding season (AF010, AF017, AF058, AF096, AM006, AM007, AM018, AM024, and AM059; Figure 5), whereas locations of four individuals indicated an avoidance of roads (i.e., further from roads) during the breeding season (AF086, AF099, AM015, and AM054; Figure 5). Four adult males showed an association with roads during the non‐breeding season (AM007, AM015, AM018, and AM024; Figure 6). Three adult males and all four females included in the non‐breeding season model exhibited an avoidance of major roads (AF017, AF056, AF058, AF086, AM006, AM054, and AM059; Figure 5). However, all confidence intervals for adult females overlapped zero, as did many of our results for the adult males, likely due to our coarse radio‐telemetry fixes which limited our inferences.

Population‐level ISSF models indicated low association with major roads during the breeding season in adult males (*β* = 4.76^−04^, 95% CI −5.42^−04^ to 1.4^−03^; Figure 6) and adult females (*β* = 5.02^−04^, 95% CI −1.52^−04^ to 1.2^−03^; Figure 6). The population‐level ISSF for adult males in the non‐breeding season showed a similar result to the breeding season (*β* = 4.83^−04^, 95% CI −1.05^−04^ to 0.1^−03^; Figure 6), whereas the adult females exhibited a lower association during the non‐breeding season (*β* = 1.9^−04^, 95% CI −9.61^−04^ to 1.3^−03^; Figure 6). However, the confidence intervals overlap the different season's point estimates, limiting our confidence in the observed reduction in association during the non‐breeding season. Similarly, the confidence interval's overlap with zero could indicate the relative lack of importance of road proximity in King Cobra movement on a population (although individual variation is clear; Figure 5).

We did not see any clear interaction between step lengths and proximity to roads (Figure S11), although males appear to slightly increase step lengths when nearer roads (*β* = 9.84^−07^, 95% CI −2.09^−06^ to 4.1^−06^) with this interaction appearing marginally greater during breeding season (*β* = 2.81^−06^, 95% CI −5.03^−06^ to 6.19^−06^). Turn angle was more similar between the sexes (Figure S12); overall showing marginally lower turn angles when closer to roads. Seasonal shifts in turn angle appear weak, or non‐existent in the case of the males.

## 4 DISCUSSION

**FIGURE 7:**
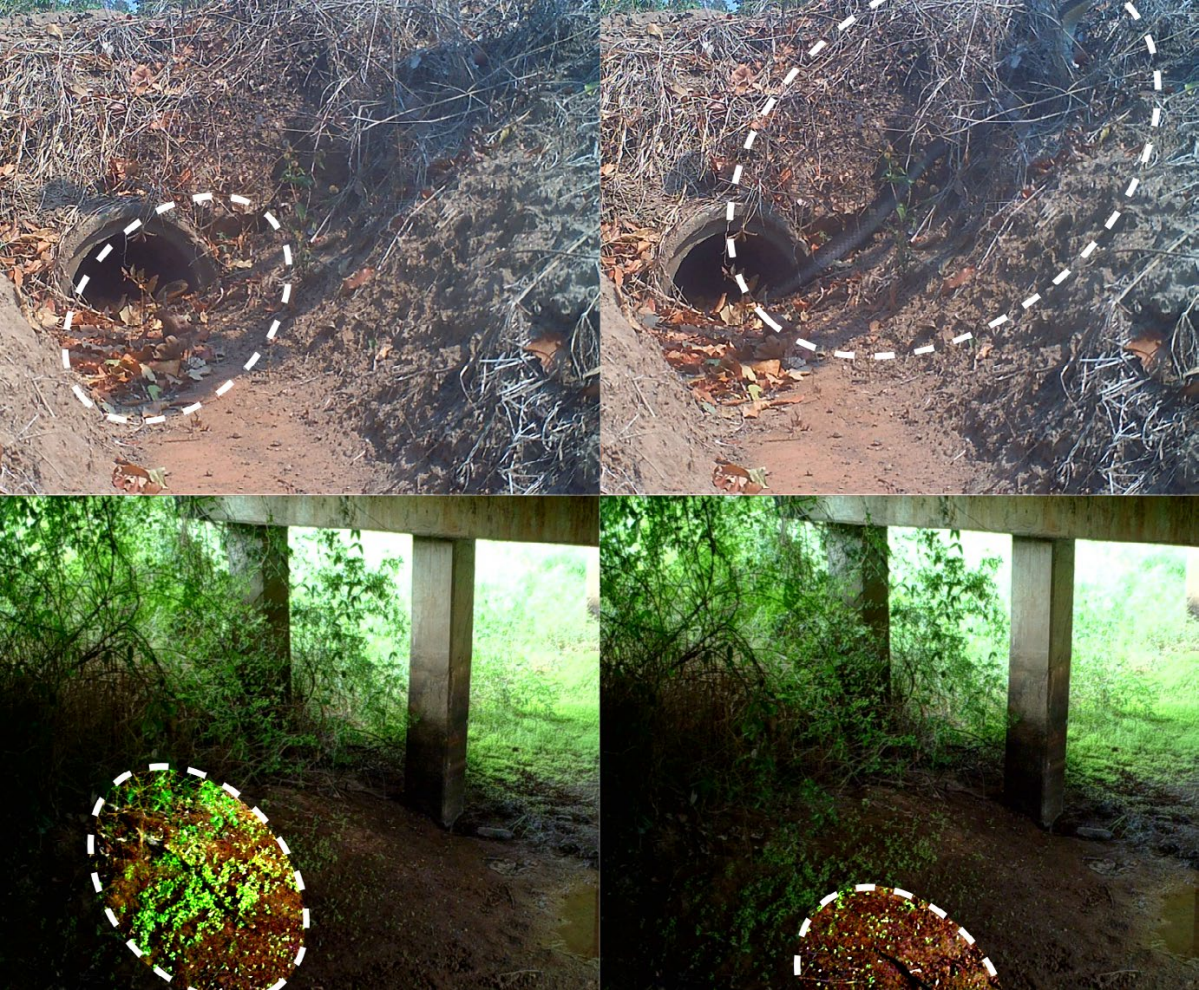
Use of road‐crossing structures by telemetered King Cobra. *Top* Use of a drainage culvert by AM015. *Bottom* Movement underneath a bridge by AM054. King Cobras in frame are highlighted with dashed white circles

We investigated the interactions of King Cobras with roads at the edge of a protected area within the Sakaerat Biosphere Reserve (SBR), Thailand. King Cobras repeatedly traversed a major four‐lane highway, with individual snakes crossing up to 37 times during the study. We documented 32 potential crossing locations within a 15.3 km stretch of highway that telemetered King Cobras could potentially use to traverse the road. We observed three telemetered King Cobras using different types of underpasses, including one drainage culvert and two bridges (evidence of two; Figure 7). All individual‐level ISSF results showed an equal or greater association with roads during the breeding season, except one individual, AM015, that we repeatedly observed moving into the forest to breed and thus further away from road structures. Our population‐level ISSF showed negligible changes in movement in relation to major roads for adult King Cobras within and outside the breeding season. However, we made direct observations of adult female King Cobras crossing a busy major road (304WNK‐NW) to access nesting sites during breeding season (Figure 4). These observations suggest that major roads bisecting typical female King Cobra occurrence distributions (within the agricultural landscape) and oviposition sites (forested areas) may present a particular mortality risk during what is already a hazardous time.

Intentional crossings structures for wildlife are absent from the SBR. We observed King Cobras in the SBR using a combination of drainage culverts and bridges to traverse the highway (Figure 4), but also attempting to cross the highway surface resulting in mortalities (Marshall et al., 2018). Because many of the telemetered King Cobras were tracked on one side of the road, and subsequently on the other side, we cannot directly confirm that individuals are routinely using underpasses as opposed to moving over the road. However, we have observed individuals moving underneath bridges (Figure 7), and we have obtained radio‐telemetry fixes directly underneath Highway 304. Highway 304 is one of the busiest roads in Thailand; in 2006, 7,488 vehicles/day were recorded (Srikrajang, 2006), which means one vehicle every 11.5 s on average. Moreover, with a consistent expansion of road infrastructure and users (Ng et al., 2020), we suspect traffic volumes to have been much higher during our study period than those observed in 2006. With all of our adult King Cobras having a total length between 2 and 4 m, it is unlikely that an individual would be able to avoid a vehicle collision during a crossing event on the road surface. Given the contrast between these risks and the relatively high rate of successful crossings, we suspect that most King Cobras actively moving over the road are at high risk of vehicle collision, and are likely to be using under‐the‐road structures as presented in this study. Additionally, our subset dBBMMs of crossing events provide strong evidence of underpass use since the only crossing attempt that was void of any crossing structure within the 99% dBBMM contour resulted in the death of a telemetered individual.

Adult males appeared to traverse the highway more frequently earlier in the year (February–May), which may be a result of mate searching behavior during the breeding season (Marshall et al., 2019; Figure 2). Other snake species have shown a propensity to cross roads more frequently during breeding periods due to increased mate searching activity (Bonnet et al., 1999; Lutterschmidt et al., 2005; Shepard et al., 2008; Sosa & Schalk, 2016). In our site, average crossing for adult males was much greater than for adult females, potentially supporting the mate searching role in road‐crossing frequency. The (marginal) increase in step lengths nearer roads during the breeding season may similarly suggest large movements (across or away from areas near roads) to areas housing females further from roads. However, this may reflect on the greater movement distance, frequency, and overall greater occurrence distributions (i.e., estimates of uncertainty surrounding movement paths in dBBMM analyses within observed tracking periods; or simply, the modelled area use of telemetered King Cobras during telemetry) of adult males at our study site (Marshall et al., 2020).

Although we are confident that our unintentional crossing structures are being used by King Cobras to facilitate movement across the road, our low sampling frequency (~1 pinpoint every 4–6 h during the day) prevented us from routinely and confidently determining the structures used for every crossing (e.g., when multiple structures are available within close proximity of each other); we only have observations of King Cobras using three of our recorded structures. By inspecting our dBBMM subsets of crossing events, it is difficult to ascertain underpass use depending on individual, location, and time of year. Figures S4–S7, for example, provide strong evidence of specific underpass use; however, Figures S8–S9 present ambiguous results due to individuals moving along the edge of the road prior to crossing. Such movements along the roadside could be driving the, albeit small, decrease in turn angles when closer to roads observed at a population level. Additionally, we performed a single evaluation of each structure (haphazard temporal sampling) that did not allow us to detect seasonal changes in characteristics; for example, habitat connectivity and vegetation cover.

Our recursive analysis presented us with approximate dates and times that each road‐crossing event occurred, and we subsequently investigated the points directly before and after a crossing in an attempt to discover if unintentional crossing structures were being used, and if so, which ones (Figure 3). Within the agricultural landscape of our study site, our results suggest that King Cobras used two bridges most frequently to cross the road (only one direct observation was made; Figure 7). The bridges are constructed over irrigation canals; the canals present an important landscape feature that appears to facilitate King Cobra movement throughout the agricultural landscape (Marshall et al., 2020). Research on other taxa suggests that the surrounding landscape provides the best predictor for which crossing structures are used over structural design and dimensions of culverts (Clevenger et al., 2002; Rodríguez et al., 1996; Yanes et al., 1995); however, we were unable to perform such analysis here due to the uncertainty surrounding if structures are actually being used. Therefore, it is difficult to determine whether King Cobras are selecting road‐crossing locations based on crossing structures presence (and the characteristics of the crossing structures), or if the landscape structure is funneling individuals to these areas (i.e., connected to established movement corridors such as irrigation canals).

While encouraging, potential King Cobra use of road‐crossing structures does not mitigate all Highway 304 potential impacts. This reflects results from other studies which suggest that the presence of road‐crossing structures (void of directional fencing, mirroring the unintentional structures in our study) has little tangible impact on roadkill effects (Cunnington et al., 2014; Rytwinski et al., 2016). Throughout our study, we have encountered seven incidents of King Cobra road mortality, five of which occurred on Highway 304. Out of these five highway mortalities, two were juvenile males, two were young of the year, and one was a telemetered adult male. The newly hatched and juvenile snakes may be less acclimated to the presence of the crossing structures, and distances between underpasses would be relatively greater and more challenging for smaller snakes to access, therefore potentially increasing juvenile snakes’ vulnerability to road mortality. Several studies have reported increased road mortalities during juvenile emergence and dispersal (Erritzoe et al., 2003; Grilo et al., 2009; Kowalczyk et al., 2009). The discovery of our telemetered adult male, AM015, was worrying, particularly as our dBBMM subsets and individual movement trajectory suggested that AM015 crossed underneath the same bridge on seven different occasions, showing a capacity to safely traverse the road, yet he crossed over the highway at least once and it led to the death of the individual (Figure 3).

Reliance on underpasses may be insufficient to reduce road mortality; for small secretive taxa (such as amphibians), fencing and directive infrastructure are required to bolster underpasses’ effectiveness (Rytwinski et al., 2016). Rytwinski et al. (2016) observations suggest that a combined approach of directive structures (e.g., fencing) and wildlife road crossings would better facilitate road‐crossing events for species that are at an increased risk of road mortality. However, King Cobras would require considerably more robust fencing than amphibians, particularly considering our personal observations of King Cobras clearing drift fences in excess of 100 cm high, alongside regular observations of arboreal behavior exhibited by telemetered King Cobras.

Female individuals of threatened taxa often require unique resources for reproduction (Brown & Weatherhead, 1997; Roth & Greene, 2006). Female King Cobras invest heavily in maternal care of eggs during oviposition and incubation (Dolia, 2018; Hrima et al., 2014; Whitaker et al., 2013), and our long‐term observations of King Cobra movement suggest that females shift their space use during the nesting season to find suitable locations for oviposition (Marshall et al., 2019, 2020). We have observed female King Cobras remaining with the nest post‐laying, which has been commonly observed in other areas of their range (Dolia, 2018; Hrima et al., 2014; Whitaker et al., 2013). Individual activity spikes (quantified by motion variance values from dynamic Brownian Bridge Movement Model output) were associated with King Cobra reproductive behaviors (i.e., oviposition site selection and nest guarding) at our site (Marshall et al., 2019). Female King Cobras in Thailand may travel into forested areas for nesting resources (e.g., substrate for nest building, vegetative cover and protection) unavailable in the agricultural matrix. Our results suggest that there are greater road mortality risks to reproductive female King Cobras during the pre‐ and post‐nesting period (Marshall et al., 2020), when individual females ordinarily using agricultural areas make large, direct moves to forested areas in order to locate oviposition sites; typically putting these individuals at greater risk of encountering major roads (Figure 4).

Unintentional crossing structures (bridges and drainage culverts) appear to facilitate King Cobra movements across a fragmented landscape, providing some promise for the survival of the population in the presence of sizable human‐made barriers like major roads. We have observed adult females moving beneath a bridge, allowing for safe passage across the 304WNK‐NW road, but we have also observed females moving over the road surface, narrowly escaping oncoming vehicles. Allocation of designed wildlife crossing structures along both of our sampled roads, using guidance from our movement data and previously outlined mortality hotspots in Silva et al. (2020), could provide a foundation for plans to reduce mortality caused by these roads for a diversity of taxa within the Sakaerat Biosphere Reserve.

## 5 CONCLUSION

Our findings add to a growing collection of road ecology literature attempting to decipher how animals interact with anthropogenic obstacles. Unintentional ecological underpasses are likely providing some level of permeability and prevent complete habitat fragmentation, which is particularly important for snakes given their reluctance to cross roads and their vulnerability when doing so (Andrews & Gibbons, 2005; Shine et al., 2004; Siers et al., 2014). King Cobras being larger and ranging further than most other reptiles make them additionally vulnerable to habitat fragmentation and the dangers of roads (Bonnet et al., 1999; Rytwinski & Fahrig, 2012). The presence of crossing structures (drainage culverts and bridges) along a major four‐lane highway appears to enable King Cobras to traverse the road, providing a level of permeability. Despite this, we continue to discover individuals that have died due to vehicle collision on Highway 304. We suggest two main future study avenues to be explored at the Sakaerat Biosphere Reserve. First, a monitoring study should be designed to evaluate the true use of unintentional wildlife crossing structures, as presented in this study, either via strategic and coordinated camera traps, or via more advanced systems, such as PIT‐tag readers (Bateman et al., 2017), or radio‐frequency identification system (RFID; Rafiq et al., 2021). Second, research is needed to evaluate whether guidance fencing combined with a Before After Control Impact (BACI) along the Highway 304 structures could aid in limiting road mortalities for both King Cobras and other terrestrial species.

## CONFLICT OF INTEREST

We declare that there are no conflicts of interest.

## AUTHOR CONTRIBUTIONS

**Max Dolton Jones:** Conceptualization (equal); Data curation (equal); Formal analysis (equal); Funding acquisition (lead); Investigation (lead); Methodology (equal); Project administration (equal); Resources (equal); Software (equal); Supervision (equal); Validation (equal); Visualization (lead); Writing – original draft (lead); Writing – review & editing (lead). **Benjamin Michael Marshall:** Conceptualization (equal); Data curation (equal); Formal analysis (equal); Investigation (equal); Methodology (equal); Software (equal); Validation (equal); Visualization (equal); Writing – review & editing (equal). **Samantha Nicole Smith:** Conceptualization (equal); Data curation (equal); Formal analysis (equal); Investigation (equal); Methodology (equal); Software (equal); Writing – review & editing (equal). **Matthew Crane:** Investigation (equal); Writing – review & editing (equal). **Inês Silva:** Investigation (equal); Writing – review & editing (equal). **Taksin Artchawakom:** Supervision (equal); Writing – review & editing (equal). **Pongthep Suwanwaree:** Funding acquisition (equal); Supervision (equal); Writing – review & editing (equal). **Surachit Waengsothorn:** Supervision (equal); Writing – review & editing (equal). **Wolfgang Wüster:** Writing – review & editing (equal). **Matt Goode:** Writing – review & editing (equal). **Colin Thomas Strine:** Conceptualization (equal); Funding acquisition (equal); Investigation (equal); Methodology (equal); Supervision (equal); Writing – review & editing (equal).

### OPEN RESEARCH BADGES

This article has been awarded Open Materials, Open Data Badges. All materials and data are publicly accessible via the Open Science Framework at https://www.movebank.org/cms/webapp?gwt_fragment=page=studies,path=study1649411628; https://zenodo.org/record/5148436#.Ygt_T‐rP02w; https://doi.org/10.5281/zenodo.5148436.

## Supporting information

Supplementary Material

## Data Availability

All data used in analyses are available on Zenodo (https://doi.org/10.5281/zenodo.5148436), including R scripts, telemetry data, and shapefiles. King Cobra telemetry data used in this study are also available via Movebank (Movebank ID: 1649411628).
